# Agreement Between Parental Self‐Reported Antiseizure Medication Use and Dispensed Prescription Records From a National Prescription Database

**DOI:** 10.1002/pds.70139

**Published:** 2025-03-25

**Authors:** Emilie Willoch Olstad, Hedvig Nordeng, Marte‐Helene Bjørk, Kaja Kristine Selmer, Kristina Gervin

**Affiliations:** ^1^ PharmacoEpidemiology and Drug Safety Research Group, Department of Pharmacy Faculty of Mathematics and Natural Sciences, University of Oslo Oslo Norway; ^2^ UiORealArt Convergence Environment University of Oslo Oslo Norway; ^3^ Department of Child Health and Development Norwegian Institute of Public Health Oslo Norway; ^4^ Department of Neurology Haukeland University Hospital Bergen Norway; ^5^ Department of Clinical Neurophysiology Haukeland University Hospital Bergen Norway; ^6^ Department of Research and Innovation, Division of Clinical Neuroscience Oslo University Hospital Oslo Norway; ^7^ National Centre for Epilepsy Oslo University Hospital Oslo Norway

**Keywords:** antiseizure medications, exposure misclassification, pregnancy, teratology, validity

## Abstract

**Purpose:**

Accurate measurement of medication exposure is crucial for studying the safety of antiseizure medications (ASMs) during pregnancy. Pregnancy safety studies of ASMs frequently rely on secondary data from drug prescription registries to assess potential teratogenic effects and impact on fetal development. This study aimed to evaluate the agreement between dispensed prescriptions registered in a national database and self‐reported ASM use by parents.

**Methods:**

The Norwegian Prescription Database (NorPD) was linked to the Norwegian Mother, Father, and Child Cohort Study (MoBa) and the Medical Birth Registry of Norway (MBRN). Participants included mothers and fathers in the MoBa‐study between 2004 and 2009. Agreement between dispensed ASM prescriptions and self‐reported use was assessed by calculating Cohen's kappa (*κ*), sensitivity, and specificity, with self‐reported use as the reference standard.

**Results:**

A total of 40 632 pregnant women and 42 247 fathers were included. Maternal dispensed ASM prescriptions during pregnancy showed strong overall agreement (*κ* = 0.81) with self‐reported use, with a sensitivity of 80.6% and specificity of 99.9%. Paternal dispensed ASM prescriptions up to 7 months prior to conception also demonstrated strong agreement (*κ* = 0.81) with self‐reported use. Analysis of individual ASMs revealed varying reliability: levetiracetam and lamotrigine had the highest agreement among maternal (*κ* = 0.92) and paternal (*κ* = 0.92) dispensed prescriptions, respectively.

**Conclusion:**

There is strong agreement between dispensed ASM prescriptions and self‐reported medication use by parents, supporting the use of prescription data for evaluating the risks of ASM use during pregnancy.

**Summary**

- Strong agreement between dispensed ASM prescriptions from NorPD and parental self‐reports in MoBa (*κ* = 0.81), validating prescription records as reliable for assessing ASM use during pregnancy.
- High sensitivity and specificity in parental self‐reports highlight the precision of self‐reported ASM data.
- Variability in reporting accuracy across ASMs, with notable agreement for levetiracetam and lamotrigine.
- Sensitivity and specificity parameters can be used to inform bias analysis addressing exposure misclassification in studies of ASM in pregnancy.

## 1 Introduction

Pharmacoepidemiological studies with accurate data on parental use of antiseizure medication (ASM) are essential to evaluate teratogenic risks [1, 2, 3, 4]. Epilepsy is a prevalent neurological condition affecting individuals of reproductive age: approximately one in 250 pregnant women have epilepsy [5]. ASMs are crucial for controlling seizures, and most pregnant women with active epilepsy require daily ASMs [6]. However, the fetal safety information on most ASMs is limited.

One of the most effective ASMs is valproate [7]. Maternal valproate use during pregnancy is associated with a significantly increased risk of congenital malformations and adverse neurodevelopmental outcomes in the children [8]. These risks have led to regulatory restrictions on valproate use in child bearing age and during pregnancy [9]. Additionally, recent debates in Europe have arisen concerning the impact of paternal ASM use during the period of spermatogenesis for the unborn child. While earlier studies have not shown a significantly increased risk for neurodevelopmental outcomes in children with paternal ASM use [10, 11], newer findings from larger cohorts suggest a potential risk, leading to authorities devising precautions regarding the use of valproate by men of reproductive age [12, 13].

For observational studies to be regulatory and clinically informative, the accuracy of parental medication measures is paramount. These studies often rely on prescribed or dispensed prescription records or self‐reported information as a proxy for fetal medication exposure, with both data sources having their strengths and limitations. Prescribed prescriptions may never be picked up from the pharmacy, and dispensed prescriptions may be subject to non‐adherence, for example, if a woman discontinues treatment when she discovers that she is pregnant. Self‐report may be subject to poor recall or recall bias in retrospective studies, as well as selective reporting [14, 15]. Consequently, researchers need to pay great attention to the extent and impact of potential exposure misclassifications in observational studies.

This study aimed to assess the agreement between both maternal and paternal dispensed prescription records using data from the Norwegian Prescription Database (NorPD) linked to self‐reported ASM as reported in the Norwegian Mother, Father, and Child Cohort Study (MoBa). Establishing the reliability of dispensed parental ASM use will enhance the validity of perinatal pharmacoepidemiological safety studies. Ultimately, safety assessments of these medications during the period of spermatogenesis and pregnancy may contribute to better healthcare guidance for parents with epilepsy.

## 2 Methods

### 2.1 Study Population

This study is based on data from NorPD, the Medical Birth Registry of Norway (MBRN), and the MoBa birth cohort study. NorPD was established in 2004 and contains information on all dispensed prescription medications in Norwegian pharmacies, including data on dispensing and amount dispensed. MBRN is a national health registry containing information about all births in Norway, including data on delivery and gestational length. MoBa is an ongoing prospective birth cohort study conducted by the Norwegian Institute of Public Health (*n* = 114 500 children, *n* = 95 200 mothers, and *n* = 75 200 fathers) [16, 17]. Participants were recruited from all over Norway from 1999 to 2008, and among all women giving birth during this time, 40.6% consented to participate. The MoBa questionnaires were distributed by mail and cover a range of different topics, including questions regarding maternal and paternal health and medication use. During pregnancy and after birth, women were asked about medication use in relation to various conditions in three different questionnaires: Q1 (gestational week 15), Q3 (gestational week 30), and Q4 (6 months after delivery). Around gestational week 15, a questionnaire was also distributed to the father (paternal Q1), in which he was asked to name any medication used in the time prior to conception. The current study is based on version 12 of the quality‐assured MoBa data files released in 2020 (maternal data) and version 9 of the quality‐assured data files released in 2015 (paternal data).

The establishment of MoBa and initial data collection was based on a license from the Norwegian Data Protection Agency and approval from the Regional Committees for Medical and Health Research Ethics (REC). The MoBa cohort is currently regulated by the Norwegian Health Registry Act. All data were de‐identified, and the linking of MoBa to health registries was handled by the Norwegian Institute of Public Health and the respective registries. The present study was approved by REC South East Norway (references 421997 and 2015/442).

### 2.2 Sample Selection

**FIGURE 1:**
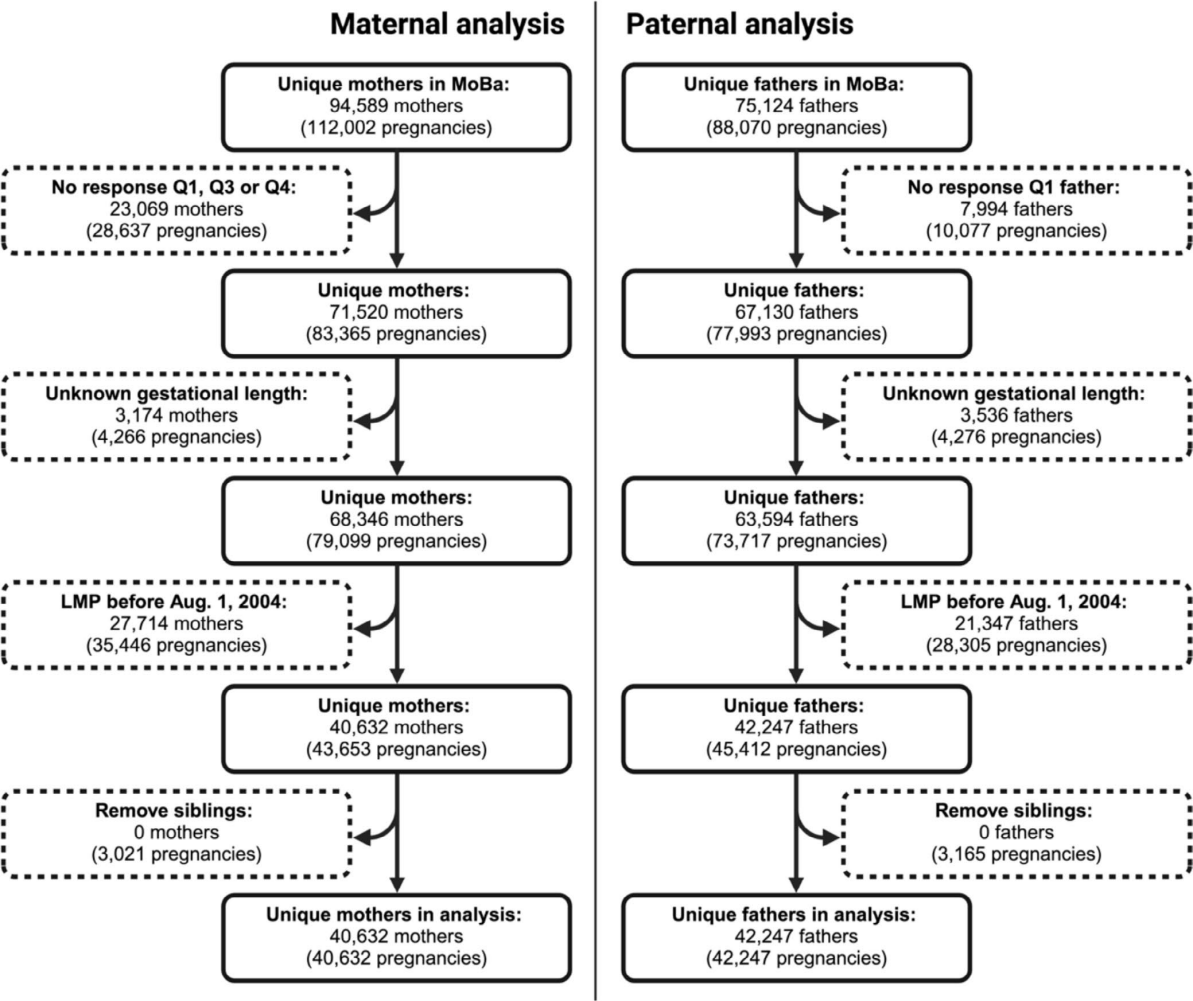
Flow diagram of the study sample selection.

The study period was January 2004 to 2009 to cover the time period of overlap between NorPD and MoBa. Mothers and fathers were selected by applying the following criteria (the number of removed pregnancies for the maternal [*n*_mat_] and paternal [*n*_pat_] analyses are indicated in parentheses; Figure 1):

- Mothers had replied to the MoBa questionnaires Q1, Q3, and Q4, and fathers had replied to the paternal Q1 questionnaire (*n*_mat_ = 28 637 and *n*_pat_ = 10 077 pregnancies were excluded).
- Pregnancies with unknown gestational length were removed as it would not be possible to approximate the first day of the last menstrual period (LMP) without this variable (*n*_mat_ = 4266 and *n*_pat_ = 4276 pregnancies were excluded).
- Pregnancies for which the LMP was earlier than August 1, 2004, were excluded, as the fathers' prescription fills should be entirely covered by NorPD, that is, 7 months before LMP, as paternal medication use in MoBa was defined as use up to 6 months before LMP, and an additional 30 days were included to account for potential carry‐over medication use (*n*_mat_ = 35 446 and *n*_pat_ = 28 305 pregnancies were excluded; Figure S1).
- For each unique parent, only one randomly selected pregnancy was included, that is, no siblings (*n*_mat_ = 3021 and *n*_pat_ = 3165 pregnancies were excluded).

The final study population included 40 632 unique women and 42 247 unique men.

### 2.3 Medication Exposure

The medications of interest in this study were ASMs. We used the World Health Organization's Anatomical Therapeutic Chemical (ATC) codes to identify ASM use in MoBa and NorPD. The code used to identify any ASM was N03. To identify individual medications, we used the sixth‐level ATC code. The ATC codes of the medications recorded used in this study were: N03AA02 (phenobarbital), N03AB02 (phenytoin), N03AE01 (clonazepam), N03AF01 (carbamazepine), N03AF02 (oxcarbazepine), N03AG01 (valproate), N03AG04 (vigabatrin), N03AX09 (lamotrigine), N03AX11 (topiramate), N03AX12 (gabapentin), N03AX14 (levetiracetam), and N03AX16 (pregabalin).

In MoBa, mothers report medication usage throughout pregnancy, in questionnaires Q1 (gestational weeks 0–13), Q3 (weeks 13–29), and Q4 (week 30 to 6 months after delivery). Hence, maternal use of medications in MoBa was defined as use during either of the time intervals corresponding to trimesters 1 (gestational weeks 0–12), 2 (weeks 13–26), or 3 (week 27 to birth). Fathers report medication use in MoBa for the time intervals 0–1 week prior to conception, 1 week–1 month prior to conception, and 1 month–6 months prior to conception. Hence, paternal medication exposure was recorded as self‐report of the medication at any of these time points.

In NorPD, medication exposure was defined as the filling of a prescription of the medication during the time interval under study (i.e., the entire pregnancy or trimester for mothers, and 6 months prior to conception for fathers). To account for potential carry‐over medication use, we allowed prescription fills prior to the time interval under study in our calculations; this was done by adding a window of 0, 30, 60, or 90 days prior to the beginning of each time interval (Figure S1). NorPD data prior to the LMP were available only for a limited number of women, and therefore, windows were only added to trimesters 2 and 3. Adding time windows is a common practice in pharmacoepidemiological studies, as the amount of medication dispensed will generally include up to a 3‐month supply of medication [18]. In the analysis of parental individual ASM use, the numbers presented cover the entire pregnancy (mothers) or the last 6 months before pregnancy (fathers).

### 2.4 Statistical Analysis

All analyses were conducted in *RStudio* (version 2023.03.1) with *R* version 4.2.3. Plots were generated with the *ggplot2* package. To examine the agreement between medication use recorded in NorPD and MoBa, we calculated Cohen's *κ*, the sensitivity, the specificity, the positive predictive value (PPV), and the negative predictive value (NPV), with self‐reports in MoBa as the reference standard. If either comparison group included less than 10 individuals, we only reported the crude numbers.

Cohen's *κ* is used to determine the agreement between categorical variables, taking into account the possibility of chance agreement [19]. Generally, 0.40 ≤ *κ* < 0.60 is considered weak agreement, 0.60 ≤ *κ* < 0.80 is considered moderate agreement, 0.80 ≤ *κ* < 0.90 is considered strong agreement, and *κ* ≥ 0.90 is considered almost perfect agreement [20]. To calculate Cohen's *κ* the “cohen. kappa()” function of the *psych* package was used.

The sensitivity, specificity, PPV, and NPV are measures of the accuracy of dispensed ASM prescriptions. The sensitivity is defined as the number of true positives divided by the sum of true positives and false negatives. The specificity is defined as the number of true negatives over the sum of true negatives and false positives. The PPV is defined as the number of true positives divided by the sum of true and false positives. The NPV is defined as the number of true negatives over the sum of the true and false negatives. To calculate these entities, self‐reported ASM use in MoBa was considered the reference standard (i.e., the “truth”). Hence, the sensitivity was calculated as recorded medication use in both MoBa and NorPD divided by the sum of these recordings and the recordings of medication use in MoBa only. Similarly, the specificity was calculated as no recorded medication use in neither MoBa nor NorPD divided by the sum of these recordings and the recordings of medication use only in NorPD. The PPV was calculated as recorded medication use in both MoBa and NorPD divided by the sum of these recordings and the recordings of medication use in NorPD only. Finally, the NPV was calculated as no recorded medication use in neither MoBa nor NorPD divided by the sum of these recordings and the recordings of medication use only in MoBa. Consequently, in our study, the sensitivity is the proportion of self‐reported use in MoBa that is also present in NorPD, the specificity is the proportion of individuals that do not self‐report ASM use in MoBa that have also not filled any ASM prescriptions recorded in NorPD, the PPV is the proportion of filled prescriptions recorded in NorPD that are also self‐reported in MoBa, and the NPV is the proportion of individuals that has not filled a prescription recorded in NorPD and also not reported ASM use in MoBa.

The primary analysis allowed for the filling of an ASM prescription up to 0 and 30 days prior to the time interval for mothers and fathers, respectively, with the time interval being the entire pregnancy (for mothers) or the 6 months prior to pregnancy (for fathers). In a sensitivity analysis of maternal medication use across trimesters, we adjusted the time window to allow for the prescription filling of an ASM 0, 30, 60, or 90 days prior to trimesters 2 and 3.

## 3 Results

### 3.1 Participant Characteristics and Prevalence of Parental ASM Use During Pregnancy

**FIGURE 2:**
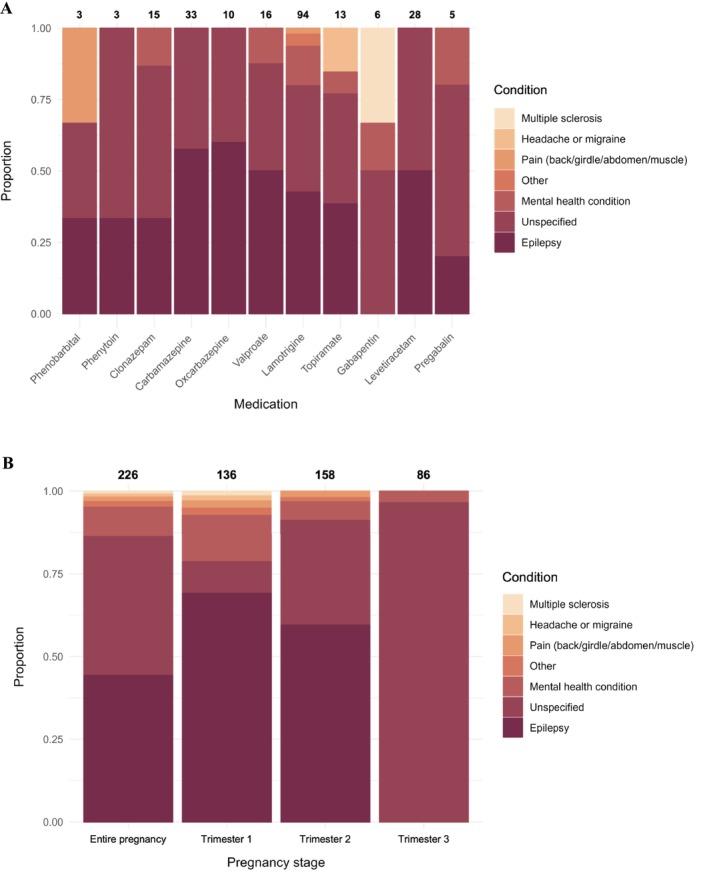
Overview of the indication for maternal ASM use as reported in MoBa: (A) for different ASMs and (B) for any ASMs in the entire pregnancy and per trimester. The number of individuals is indicated above the respective bars. Which conditions constitute the “Other” category is outlined in Table S1. Note that the total number of exposed women per medication group may add up to more than the total numbers of ASM users indicated in Table 1, as women using a medication on several indications will be counted multiple times in the above plots.

The study included pregnant women (*n* = 40 632) and fathers (*n* = 42 247) from the MoBa cohort (Figure 1), of which 0.3% (*n* = 139) and 0.5% (*n* = 202) reported using ASMs, respectively. The mean age of participants was 30.5 ± 5.5 years for mothers and 33.4 ± 5.5 years for fathers. A significant proportion of the participants reporting ASM use in MoBa had a reported history of epilepsy (69.8% of mothers and 65.2% of fathers), while others were prescribed ASMs for different indications such as mental health or pain conditions (Figure 2). The most common ASMs reported by the mothers included lamotrigine (*n* = 64), carbamazepine (*n* = 21), levetiracetam (*n* = 18), valproate (*n* = 13), clonazepam (*n* = 11), and topiramate (*n* = 10; Table 1). Of note, a large proportion of the mothers reported using ASMs for unspecified conditions in trimesters 2 and 3, where epilepsy is not specifically included as an indication in the corresponding questionnaires (Figure 2A). Similarly, among fathers, lamotrigine (*n* = 60) was the most commonly reported medication, followed by carbamazepine and valproate (*n* = 52 each), and clonazepam (*n* = 12; Table 2).

**TABLE 1:** Overview of maternal use of antiseizure medications during pregnancy, stratified by medication name (*n* = 40 632).

	Medication exposed mothers		Agreement	Overlap between MoBa and NorPD				Validity			
	MoBa (*n*)	NorPD (*n*)	Cohen's *κ* (95% C.I.)	Not MoBa, not NorPD (*n*)	Not MoBa, NorPD (*n*)	MoBa, not NorPD (*n*)	MoBa, NorPD (*n*)	Sensitivity^a^ (95% C.I.)	Specificity^b^ (95% C.I.)	PPV^c^ (95% C.I.)	NPV^d^ (95% C.I.)
Any ASM	139	138	0.81 (0.76–0.86)	40 467	26	27	112	80.6 (73.2–86.3)	99.9 (99.9–100)	81.2 (73.8–86.8)	99.9 (99.9–100)
Clonazepam	11	13	0.75 (0.56–0.94)	40 617	4	2	9	81.8 (52.3–94.9)	100 (100–100)	69.2 (42.4–87.3)	100 (100–100)
Carbamazepine	21	21	0.90 (0.81–1)^e^	40 609	2	2	19	90.5 (71.1–97.4)	100 (100–100)	90.5 (71.1–97.4)	100 (100–100)
Valproate	13	10	0.87 (0.72–1)^e^	40 619	0	3	10	76.9 (49.7–91.8)	100 (100–100)	100 (72.3–100)	100 (100–100)
Lamotrigine	64	70	0.81 (0.73–0.88)	40 552	16	10	54	84.4 (73.6–91.3)	100 (99.9–100)	77.1 (66.1–85.4)	100 (100–100)
Levetiracetam	18	21	0.92 (0.84–1)^e^	40 611	3	0	18	100 (82.4–100)	100 (100–100)	85.7 (65.4–95.0)	100 (100–100)

*Note:* Drug dispensations between the LMP and birth were included. The analysis includes pregnant women with LMP after August 1, 2004. Calculations were not performed for ASMs with less than 10 exposures.

Abbreviations: ASM: antiseizure medication; C.I.: confidence interval; LMP: last menstrual period; MoBa: the Norwegian Mother, Father, and Child Birth Cohort; NorPD: the Norwegian Prescription Database; NPV: negative predictive value; PPV: positive predictive value.

^a^ Sensitivity = TP/(TP + FN) = MoBa and NorPD/(MoBa and NorPD + MoBa, not NorPD).

^b^ Specificity = TN/(TN + FP) = Not MoBa, not NorPD/(not MoBa, not NorPD + not MoBa, NorPD).

^c^ PPV = TP/(TP + FP) = MoBa and NorPD/(MoBa and NorPD + not MoBa, NorPD).

^d^ NPV = TN/(TN + FN) = Not MoBa, not NorPD/(not MoBa, not NorPD + MoBa, not NorPD).

^e^ The lower and upper C.I.s exceed the absolute value of 1 and are set to ±1.

**TABLE 2:** Overview of paternal use of antiseizure medications during pregnancy, stratified by medication name (*n* = 42 247).

	Medication exposed fathers		Agreement	Overlap between MoBa and NorPD				Validity			
	MoBa (*n*)	NorPD (*n*)	Cohen's *κ* (95% C.I.)	Not MoBa, not NorPD (*n*)	Not MoBa, NorPD (*n*)	MoBa, not NorPD (*n*)	MoBa, NorPD (*n*)	Sensitivity^a^ (95% C.I.)	Specificity^b^ (95% C.I.)	PPV^c^ (95% C.I.)	NPV^d^ (95% C.I.)
Any ASM	202	242	0.81 (0.77–0.85)	41 983	62	22	180	89.1 (84.1–92.7)	99.9 (99.8–99.9)	74.4 (68.5–79.5)	100 (99.9–100)
Clonazepam	12	15	0.44 (0.21–0.68)	42 226	9	6	6	50.0 (25.4–74.6)	100 (100–100)	40.0 (19.8–64.3)	100 (100–100)
Carbamazepine	52	59	0.83 (0.75–0.90)	42 182	13	6	46	88.5 (77.0–94.6)	100 (100–100)	78.0 (65.9–86.7)	100 (100–100)
Valproate	52	52	0.87 (0.80–0.94)	42 188	7	7	45	86.5 (74.7–93.3)	100 (100–100)	86.5 (74.7–93.3)	100 (100–100)
Lamotrigine	60	66	0.92 (0.87–0.97)	42 179	8	2	58	96.7 (88.6–99.1)	100 (100–100)	87.9 (77.9–93.7)	100 (100–100)

*Note:* Father's reported medication use in MoBa up to 6 months prior to pregnancy are included, dispensed prescription up to 30 days prior to the 6 months are included to account for carry‐over drug utilization. The analyses were restricted to fathers with partners having their LMP after August 1, 2004. Calculations were not performed for ASMs with less than 10 exposures.

Abbreviations: ASM: antiseizure medications; C.I.: confidence interval; LMP: last menstrual period; MoBa: the Norwegian Mother, Father, and Child Birth Cohort; NorPD: the Norwegian Prescribed Drug Registry; NPV: negative predictive value; PPV: positive predictive value.

^a^ Sensitivity = TP/(TP + FN) = MoBa and NorPD/(MoBa and NorPD + MoBa, not NorPD).

^b^ Specificity = TN/(TN + FP) = Not MoBa, not NorPD/(not MoBa, not NorPD + not MoBa, NorPD).

^c^ PPV = TP/(TP + FP) = MoBa and NorPD/(MoBa and NorPD + not MoBa, NorPD).

^d^ NPV = TN/(TN + FN) = Not MoBa, not NorPD/(not MoBa, not NorPD + MoBa, not NorPD).

### 3.2 Comparison of Dispensed Prescription and Self‐Reported Data on Maternal ASM Use

We first assessed the agreement between maternal prescription records from NorPD with self‐reported use in MoBa for any ASM (classified under the ATC code N03). By calculating Cohen's kappa coefficient (*κ*) we evaluated the overall agreement between NorPD and MoBa, and the sensitivity, specificity, PPV, and NPV were used to assess the validity of NorPD prescription records using self‐reports in MoBa as the reference. Cohen's *κ* provided an adjusted measure of agreement, taking into account the possibility of chance agreement, while sensitivity, specificity, PPV, and NPV offered insights into the proportion of MoBa self‐reports and non‐reports overlapping with NorPD, respectively.

An overview of maternal ASM use, stratified by the timing of use (any time during pregnancy or per specific trimester) and the prescription fill date relative to the pregnancy or trimester (i.e., 0 days, or 30, 60, or 90 days before pregnancy or the start of each trimester), is presented in Table S2. This analysis revealed a strong overall agreement between dispensed prescriptions and self‐reports (*κ* = 0.81) when calculated for any time during pregnancy. However, some variations when the agreement was calculated per trimester were observed. The second trimester showed a strong agreement (*κ* > 0.82).

The sensitivity during the entire pregnancy was 80.6%, indicating that a high proportion of self‐reported ASM use in MoBa is confirmed by prescription fills in NorPD. Similarly, the PPV during the entire pregnancy was 81.2%, indicating that most of the ASM prescriptions filled in NorPD were also self‐reported in MoBa. Both the specificity and NPV were 99.9%, indicating that when mothers do not report using an ASM during pregnancy, it is rare to find a corresponding dispensed prescription in NorPD (occurred in 26 women), and conversely, few women self‐report ASM use in MoBa when there is no prescription fill recorded in NorPD (occurred in 27 women). The trends in sensitivity and PPV aligned with the agreement findings, with the second trimester showing the highest sensitivity and PPV (> 83.2% and > 80.0%, respectively; Table S2). Notably, while the sensitivity was also relatively high in the third trimester (> 73.3%), the PPV in the third trimester was lower than in the first trimester (> 48.2% vs. > 75.8%, respectively).

Table 1 presents a detailed breakdown of the agreement and validity metrics for maternal use of individual ASMs, revealing notable differences in reporting accuracy among the different medications. For instance, levetiracetam demonstrated the highest *κ* value of 0.92, indicating an almost perfect agreement between dispensed prescriptions and self‐reported use. Clonazepam, being used for epilepsy by a limited number of patients, showed the lowest *κ* value of 0.75. It is important to note that the available numbers for some individual ASMs were small, and therefore the Cohen's *κ*, sensitivity, specificity, PPV, and NPV were not reported.

### 3.3 Comparison of Dispensed Prescription Data and Self‐Reported Paternal ASM Use

The analyses of paternal use of individual ASMs are presented in Table 2. The analysis revealed an overall strong agreement between dispensed prescription records and self‐reported ASM use by fathers (*κ* = 0.81). The sensitivity was 89.1%, showing that most self‐reports in MoBa have a corresponding prescription fill in NorPD. The PPV was 74.4%, indicating that most, but substantially fewer, filled prescriptions recorded in NorPD have also been self‐reported in MoBa. The specificity was 99.9% and the NPV was 100%.

For individual ASMs, where the numbers were sufficiently large to allow for detailed analysis, the agreement between self‐reported and prescription data was comparable to, and in some cases higher than, the maternal estimates. This is particularly noteworthy for lamotrigine, where there was an almost perfect agreement (*κ* = 0.92). Lamotrigine additionally had the highest sensitivity (96.7%) and PPV (85.7%) of the medications analyzed for the father.

## 4 Discussion

This study provides critical insights into the reliability of dispensed prescription records of parental ASM use as compared to patients' self‐reports. Our findings reveal an overall strong agreement between dispensed prescriptions and self‐reported ASM use for both mothers and fathers. This high level of concordance underscores the reliability of both prescription records and self‐reported data as sources of information for ASMs in pharmacoepidemiological studies.

The agreement between both maternal and paternal dispensed prescription records and self‐reported ASM use was moderate to almost perfect for most ASMs, indicating a high level of reliability of the prescription data for any ASM use. Notably, there was a pronounced variation in the agreement when examining individual ASMs, with levetiracetam exhibiting the highest agreement for mothers, suggesting good adherence to prescriptions of levetiracetam. This can be explained by the indications for levetiracetam use being almost exclusively for epilepsy. ASM agreement is higher for epilepsy than for other indications in pregnancy [21]. We observed some variations in the agreement across trimesters for maternal ASM use, with the second trimester showing the strongest agreement. Of note, the self‐reported data are probably most accurate for the second trimester, as the questionnaires covering this trimester were completed around weeks 18 and 30. Hence, the good agreement with prescription records for this period is reassuring.

Overall, the sensitivity of both maternal and paternal ASM use was relatively high, indicating that a large proportion of self‐reported ASM use in MoBa has corresponding prescription fills in NorPD. This suggests that there are relatively few instances where self‐reported ASM use goes unrecorded in the prescription database. Furthermore, like the agreement trend per trimester, the sensitivity was also highest in the second trimester. This trend suggests that the reliability of dispensed ASM prescriptions, as confirmed by self‐reports, varies across different stages of pregnancy. It highlights the importance of considering differential exposure misclassification across trimesters, which may impact the assessment of teratogenic risks associated with ASMs during specific periods of pregnancy.

The PPVs largely reflected the trend seen for the sensitivities, meaning that most prescription fills recorded in NorPD had a corresponding self‐report in MoBa. Notably, in the third trimester, the PPV was substantially lower than the sensitivity. This indicates that a higher proportion of self‐reports in MoBa had a corresponding filled prescription in NorPD than oppositely. This may be explained by the nature of the registry data: while the woman may have filled the prescription during the third trimester, she may not have initiated treatment until after birth.

Both the specificity and the NPV were very high for all comparisons. These entities indicate minimal false positives relative to the true negatives, and minimal false negatives relative to the true negatives, respectively. The high specificity indicates that when mothers do not report using an ASM during pregnancy, it is very rare to find a corresponding dispensed prescription in NorPD. This occurred only in 26 women and may be due to lack of recall or lack of intake of prescribed medications (i.e., non‐adherence). Importantly, however, the specificity and NPV should be interpreted with caution, considering the very small number of ASM users relative to the large number of non‐users.

The high agreement between maternal ASM prescription records and self‐reported data is particularly reassuring given the critical importance of accurate exposure classification when studying the teratogenic effects of ASMs. Our results align with previous research [18, 22], showing that prescription records are reliable sources of information about ASM use. However, the findings also highlight the variability that can exist between different medications, as noted in other studies [18]. This could be attributed to various factors such as the conditions for which they are prescribed, the public perception of the drugs, and side‐effects. Some types of ASMs, such as clonazepam for non‐epilepsy indications, could have been used “as needed,” and not used during the reporting period, even if the parent filled the prescription. Nevertheless, these results provide valuable insights into the patterns of ASM dispensed prescriptions, which could inform assessments of exposure misclassifications, for example, in probabilistic bias analysis [23]. Understanding these differences is crucial for interpreting prescription data accurately in studies that aim to capture medication use more effectively.

Determining the accuracy of paternal prescribed ASMs addresses a gap in existing research. Recent preclinical and clinical studies suggest an association between paternal valproate use during spermatogenesis and adverse child neurodevelopmental outcomes [13]. This underscores the importance of a validation study addressing paternal ASM exposure validity. The data in the present study covered fathers' medication use 6 months prior to pregnancy start and until the beginning of pregnancy. This time frame is critical, as it encompasses periods relevant for sperm development and potential conception, thereby offering insights into the preconception exposure risks. The mostly strong agreement between paternal dispensed ASM prescriptions and self‐reported ASM use strengthens the confidence in the use of dispensed prescriptions in observational studies where paternal influences on fetal and child health outcomes are of interest. The high level of agreement for valproate is especially reassuring given the extensive use of valproate prescription data to determine its teratogenic and neurotoxic risks. These results are in agreement with and a significant expansion of a previous study which investigated the agreement of any paternal ASM use during pregnancy [24].

Our findings have important implications for both research and clinical practice. From a research perspective, the demonstrated reliability of dispensed ASM prescriptions supports the continued use of data on dispensed prescriptions in pharmacoepidemiological studies. This is crucial for expanding our understanding of the effects of ASMs on child health outcomes, including the potential impacts of paternal medication use. Ultimately, safety assessments of these medications during the period of spermatogenesis and pregnancy may contribute to better healthcare guidance for parents with epilepsy or other ASM‐treated conditions, such as bipolar disorder and headache disorders.

While our study benefits from two nationwide registries and a large prospective birth cohort, it is not without limitations. We used self‐reported data as the reference standard, which may be subject to poor recall. This is most likely non‐differential as the MoBa study was prospective, and parents reported use during pregnancy. Also, maternal self‐reported ASM use in MoBa has previously been validated against objective serum concentration measurements in maternal and umbilical cords showing very good agreements. ASMs were detected in 95% of the plasma samples where the mother reported use of such medications during pregnancy [25]. Additionally, not all ASMs were used for epilepsy, and due to low numbers, we could not stratify analysis by indication. Small sample sizes can lead to less stable *κ* values and may not accurately reflect the true agreement level for some of the ASMs. Therefore, caution must be exercised in interpreting the results for ASMs with limited data. Finally, the 40% response rate of MoBa raises concerns for the generalizability of the data. However, previous studies have demonstrated that the MoBa cohort is broadly representative of the Norwegian population with respect to key health and demographic factors, which supports the validity of our findings [26].

In conclusion, this study assessed the reliability of dispensed parental ASM prescriptions against self‐reported use, finding strong agreement between the two ASM data sources. These observations underscore the reliability of maternal and paternal ASM prescription data in perinatal pharmacoepidemiological research. While acknowledging limitations such as potential poor recall and non‐adherence to prescribed ASMs, this work supports the continued practice of utilizing both prescription records and self‐reported ASM use in pharmacoepidemiological research.

### 4.1 Plain Language Summary

Accurate measurement of medication exposure is crucial for studying the safety of ASMs during pregnancy. Pharmacoepidemiological studies often use data from drug prescription registries to understand the potential risks these medications might pose to a developing fetus. Our study aimed to evaluate how well the information from a national prescription database matched the ASM use reported by parents. We analyzed data from the MoBa and compared it with records from the NorPD. Our findings revealed an overall strong agreement between dispensed ASM prescriptions from NorPD and parental self‐reports in MoBa, validating prescription records as reliable for assessing parental ASM use during pregnancy.

## Author Contributions

K.G. initiated and led the project. All authors discussed the analytical approach. E.W.O. conducted the analyses. K.G. and E.W.O. wrote the first draft of the manuscript. H.N. was responsible for the data. All authors contributed to the interpretation of the results. All authors have read and approved the final manuscript.

## Disclosure

This work is a significant expansion of a previous study, which investigated the agreement of paternal medication use, including any paternal ASM use during pregnancy, to assess the reliability of paternal medication use as negative controls in pregnancy medication safety studies.

## Ethics Statement

The present study was approved by REC South East Norway (references 421997 and 2015/442).

## Conflicts of Interest

M.‐H.B. reports advisory board honoraria or/and speaking honoraria from Pfizer, Jazz Pharmaceuticus, Angelini Pharma, AbbVie, Lundbeck and Eisai. Received biostatistical research support from Novartis unrelated to the present study; her department has received funding from valproate marked authorization holders to conduct postmarketing drug safety research outside the submitted work, and she is PI on an industry financed phase IV study of a migraine preventive drug (eptinezumab) unrelated to the drugs studied in the present work. K.K.S. has received consultant and speaker's honoraria from Roche and OrionPharma. She also received non‐personal sponsorships from Desitin and Eisai AB in relation to organizing conferences. The other authors declare no conflicts of interest.

## Supporting information


Table S1.
Table S2.

## References

[1] J. W. Dreier , J. Christensen , J. Igland , et al., “Prenatal Exposure to Antiseizure Medications and Risk of Epilepsy in Children of Mothers With Epilepsy,” JAMA Network Open 7 (2024): e2356425. 38407908 10.1001/jamanetworkopen.2023.56425 PMC10897746

[2] J. M. Cohen , S. Alvestad , E. A. Suarez , et al., “Comparative Risk of Major Congenital Malformations With Antiseizure Medication Combinations vs Valproate Monotherapy in Pregnancy,” Neurology 102 (2024): e207996, 10.1212/WNL.0000000000207996. 38165339 PMC10870741

[3] J. M. Cohen , S. Alvestad , C. E. Cesta , et al., “Comparative Safety of Antiseizure Medication Monotherapy for Major Malformations,” Annals of Neurology 93 (2023): 551–562, 10.1002/ana.26561. 36433783

[4] J. Christensen , B. B. Trabjerg , Y. Sun , et al., “Prenatal Exposure to Valproate and Risk of Congenital Malformations‐Could We Have Known Earlier?‐A Population‐Based Cohort Study,” Epilepsia 62 (2021): 2981–2993, 10.1111/epi.17085. 34585373

[5] R. Bromley , J. Weston , N. Adab , et al., “Treatment for Epilepsy in Pregnancy: Neurodevelopmental Outcomes in the Child,” Cochrane Database of Systematic Reviews 2014, no. 10 (2014): 42, 10.1002/14651858.CD010236.pub2. PMC7390020 25354543

[6] R. Charlton , E. Garne , H. Wang , et al., “Antiepileptic Drug Prescribing Before, During and After Pregnancy: A Study in Seven European Regions,” Pharmacoepidemiology and Drug Safety 24 (2015): 1144–1154, 10.1002/pds.3847. 26272314

[7] A. Marson , G. Burnside , R. Appleton , et al., “The SANAD II Study of the Effectiveness and Cost‐Effectiveness of Valproate Versus Levetiracetam for Newly Diagnosed Generalised and Unclassifiable Epilepsy: An Open‐Label, Non‐Inferiority, Multicentre, Phase 4, Randomised Controlled Trial,” Lancet 397 (2021): 1375–1386, 10.1016/S0140-6736(21)00246-4. 33838758 PMC8047813

[8] T. Tomson , D. Battino , E. Bonizzoni , et al., “Comparative Risk of Major Congenital Malformations With Eight Different Antiepileptic Drugs: A Prospective Cohort Study of the EURAP Registry,” Lancet Neurology 17 (2018): 530–538, 10.1016/S1474-4422(18)30107-8. 29680205

[9] O. A. Hope and K. M. Harris , “Management of Epilepsy During Pregnancy and Lactation,” BMJ 382 (2023): e074630, 10.1136/bmj-2022-074630. 37684052

[10] T. Tomson , G. Muraca , and N. Razaz , “Paternal Exposure to Antiepileptic Drugs and Offspring Outcomes: A Nationwide Population‐Based Cohort Study in Sweden,” Journal of Neurology, Neurosurgery, and Psychiatry 91 (2020): 907–913, 10.1136/jnnp-2020-323028. 32651245

[11] G. Veiby , A. K. Daltveit , S. Schjølberg , et al., “Exposure to Antiepileptic Drugs In Utero and Child Development: A Prospective Population‐Based Study,” Epilepsia 54 (2013): 1462–1472, 10.1111/epi.12226. 23865818 PMC3766256

[12] D. Ibi , Y. Fujiki , N. Koide , G. Nakasai , R. Takaba , and M. Hiramatsu , “Paternal Valproic Acid Exposure in Mice Triggers Behavioral Alterations in Offspring,” Neurotoxicology and Teratology 76 (2019): 106837, 10.1016/j.ntt.2019.106837. 31654689

[13] “EMA Review of Data on Paternal Exposure to Valproate, European Medicines Agency, 2024.”

[14] A. Althubaiti , “Information Bias in Health Research: Definition, Pitfalls, and Adjustment Methods,” Journal of Multidisciplinary Healthcare 9 (2016): 211–217, 10.2147/JMDH.S104807. 27217764 PMC4862344

[15] P. H. Beardon , M. M. McGilchrist , A. D. McKendrick , D. G. McDevitt , and T. M. MacDonald , “Primary Non‐Compliance With Prescribed Medication in Primary Care,” BMJ 307 (1993): 846–848, 10.1136/bmj.307.6908.846. 8401129 PMC1678870

[16] P. Magnus , L. M. Irgens , K. Haug , et al., “Cohort Profile: The Norwegian Mother and Child Cohort Study (MoBa),” International Journal of Epidemiology 35 (2006): 1146–1150, 10.1093/ije/dyl170. 16926217

[17] P. Magnus , C. Birke , K. Vejrup , et al., “Cohort Profile Update: The Norwegian Mother and Child Cohort Study (MoBa),” International Journal of Epidemiology 45, no. 2 (2016): dyw029, 10.1093/ije/dyw029. 27063603

[18] S. Skurtveit , R. Selmer , I. Odsbu , and M. Handal , “Self‐Reported Data on Medicine Use in the Norwegian Mother and Child Cohort Study Compared to Data From the Norwegian Prescription Database,” Norsk Epidemiologi 24 (2014): 1–2, 10.5324/nje.v24i1-2.1824.

[19] J. Cohen , “A Coefficient of Agreement for Nominal Scales,” Educational and Psychological Measurement 20 (1960): 37–46, 10.1177/001316446002000104.

[20] M. L. McHugh , “Interrater Reliability: The Kappa Statistic,” Biochem Med (Zagreb) 22 (2012): 276–282. 23092060 PMC3900052

[21] J. M. Cohen , R. Selmer , K. Furu , and Ø. Karlstad , “Interrupted Time Series Analysis to Assess Changes in Prescription Filling Around Conception and Implications for Exposure Misclassification,” Pharmacoepidemiology and Drug Safety 29 (2020): 745–749, 10.1002/pds.4974. 32128905

[22] A. S. Frank , A. Lupattelli , D. S. Matteson , and H. Nordeng , “Maternal Use of Thyroid Hormone Replacement Therapy Before, During, and After Pregnancy: Agreement Between Self‐Report and Prescription Records and Group‐Based Trajectory Modeling of Prescription Patterns,” Clinical Epidemiology 10 (2018): 1801–1816, 10.2147/CLEP.S175616. 30584374 PMC6283256

[23] M. E. Wood , K. L. Lapane , M. M. H. J. van Gelder , D. Rai , and H. M. E. Nordeng , “Making Fair Comparisons in Pregnancy Medication Safety Studies: An Overview of Advanced Methods for Confounding Control,” Pharmacoepidemiology and Drug Safety 27 (2018): 140–147, 10.1002/pds.4336. 29044735 PMC6646901

[24] J. M. Cohen , M. E. Wood , S. Hernandez‐Diaz , and H. Nordeng , “Agreement Between Paternal Self‐Reported Medication Use and Records From a National Prescription Database,” Pharmacoepidemiology and Drug Safety 27 (2018): 413–421, 10.1002/pds.4411. 29488294

[25] E. S. N. Husebye , B. Riedel , A.‐L. Bjørke‐Monsen , et al., “Vitamin B Status and Association With Antiseizure Medication in Pregnant Women With Epilepsy,” Epilepsia 62 (2021): 2968–2980, 10.1111/epi.17076. 34590314

[26] R. M. Nilsen , S. E. Vollset , H. K. Gjessing , et al., “Self‐Selection and Bias in a Large Prospective Pregnancy Cohort in Norway,” Paediatric and Perinatal Epidemiology 23 (2009): 597–608, 10.1111/j.1365-3016.2009.01062.x. 19840297

