# Supplementary material for: Agreement Between Parental Self‐Reported Antiseizure Medication Use and Dispensed Prescription Records From a National Prescription Database
Source: Pharmacoepidemiol Drug Saf. 2025 Mar 25;34(4):e70139. doi: 10.1002/pds.70139 (PMC11937424; doi:10.1002/pds.70139)
Supplement: Supplementary file 1 — Table S1. Table S2. [file PDS-34-e70139-s001.docx]

**Supplementary Table S1.** Overview of conditions part of the “Other” category in Figures 1 and 2, and the number of times this indication was reported.

| **Condition** | **# reported ASM** |  |
| --- | --- | --- |
| Nausea/vomiting | 4 |  |
| Vaginal or bladder condition | 3 |  |
| Fatigue/sleeping problem | 2 |  |
| Allergy | 1 |  |

**Supplementary Table S2.** Overview of maternal use of antiseizure medications during pregnancy, stratified by time of use (all pregnancy or specific trimesters) and whether the filled prescription should be within the days of pregnancy (i.e., 0 days) or 30, 60 or 90 days before pregnancy/trimester. The data were restricted to mothers having their last menstrual period before pregnancy after August 1, 2005. There were 40,632 mothers in total.

|  | **Prevalence of medication** | | **Agreement** | **Overlap between MoBa and NorPD** | | | | **Validity** | |
| --- | --- | --- | --- | --- | --- | --- | --- | --- | --- |
|  | **MoBa**  **(*n*)** | **NorPD**  **(*n*)** | **Cohen’s *κ***  **(95% C.I.)** | **Not MoBa, not NorPD (*n*)** | **Not MoBa, NorPD (*n*)** | **MoBa, not NorPD (*n*)** | **MoBa, NorPD (*n*)** | **Sensitivity***  **(95% C.I.)** | **Specificity****  **(95% C.I.)** |
| **Any time during preg.**  *0 days bef. preg.*  *30 days bef. preg.*  *60 days bef. preg.*  *90 days bef. preg.* | 139  139  139  139 | 138  138  138  139 | 0.81 (0.76–0.86)  0.81 (0.76–0.86)  0.81 (0.76–0.86)  0.81 (0.75–0.86) | 40,467  40,467  40,467  40,466 | 26  26  26  27 | 27  27  27  27 | 112  112  112  112 | 80.6 (73.2–86.3)  80.6 (73.2–86.3)  80.6 (73.2–86.3)  80.6 (73.2–86.3) | 99.9 (99.9–100)  99.9 (99.9–100)  99.9 (99.9–100)  99.9 (99.9–100) |
| **Trimester 1 only**  *0 days bef. tri. 1*  *30 days bef. tri. 1*  *60 days bef. tri. 1*  *90 days bef. tri. 1* | 120  120  120  120 | 97  98  98  99 | 0.69 (0.62–0.76)  0.69 (0.62–0.76)  0.69 (0.62–0.76)  0.68 (0.61–0.75) | 40,490  40,489  40,489  40,488 | 22  23  23  24 | 45  45  45  45 | 75  75  75  75 | 62.5 (53.6–70.7)  62.5 (53.6–70.7)  62.5 (53.6–70.7)  62.5 (53.6–70.7) | 100 (99.9–100)  99.9 (99.9–100)  99.9 (99.9–100)  99.9 (99.9–100) |
| **Trimester 2 only**  *0 days bef. tri. 2*  *30 days bef. tri. 2*  *60 days bef. tri. 2*  *90 days bef. tri. 2* | 101  101  101  101 | 103  110  113  115 | 0.82 (0.77–0.88)  0.84 (0.79–0.90)  0.85 (0.80–0.90)  0.85 (0.80–0.90) | 40,512  40,510  40,509  40,508 | 19  21  22  23 | 17  12  10  9 | 84  89  91  92 | 83.2 (74.7–89.2)  88.1 (80.4–93.1)  90.1 (82.7–94.5)  91.1 (83.9–95.2) | 100 (99.9–100)  100 (99.9–100)  100 (99.9–100)  99.9 (99.9–100) |
| **Trimester 3 only**  *0 days bef. tri. 3*  *30 days bef. tri. 3*  *60 days bef. tri. 3*  *90 days bef. tri. 3* | 75  75  75  75 | 114  122  128  134 | 0.58 (0.50–0.66)  0.61 (0.53–0.69)  0.63 (0.55–0.71)  0.65 (0.57–0.73) | 40,498  40,495  40,493  40,491 | 59  62  64  66 | 20  15  11  7 | 55  60  64  68 | 73.3 (62.4–82.0)  80.0 (69.6–87.5)  85.3 (75.6–91.6)  90.7 (82.0–95.4) | 99.9 (99.8–99.9)  99.9 (99.8–99.9)  99.8 (99.8–99.9)  99.8 (99.8–99.9) |

**Abbreviations:** bef.: before; C.I.: confidence interval; NorPD: the Norwegian Prescribed Drug Registry; MoBa: the Norwegian Mother, Father and Child Birth Cohort; preg.: pregnancy; tri.: trimester.

* Sensitivity = TP/(TP+FN) = MoBa and NorPD / (MoBa and NorPD + MoBa, not NorPD)

** Specificity = TN/(TN+FP) = Not MoBa, not NorPD / (not MoBa, not NorPD + not MoBa, NorPD
